# Root tragedy of the commons: Revisiting the mechanisms of a misunderstood theory

**DOI:** 10.3389/fpls.2022.960942

**Published:** 2022-08-04

**Authors:** Ciro Cabal

**Affiliations:** ^1^High Meadows Environmental Institute, Princeton University, Princeton, NJ, United States; ^2^Department of Biogeography and Global Change, National Museum of Natural Sciences, MNCN, CSIC, Madrid, Spain

**Keywords:** game theory, plant behavioral ecology, plant competition, plant interaction mechanisms, root foraging strategies, root methods

## Abstract

Fine root density in the soil is a plant functional trait of paramount importance for plant ecology and agriculture. Fine root proliferation by plants involves complex plant strategies that may depend on various abiotic and biotic factors. Concretely, the root tragedy of the commons (RToC) is a behavioral strategy predicted by game theory models in which interacting plants forage for soil resources inefficiently. Generally, researchers assume that the RToC is a proactive competition strategy directly induced by the non-self roots. In this opinion, I recall Hardin’s original definition of the tragedy of the commons to challenge this notion. I argue that the RToC is a suboptimal phenotypically plastic response of the plants based on the soil resource information exclusively, and I discuss how this alternative perspective carries important implications for the design of experiments investigating the physiological mechanisms underlying observable plant root responses.

## Introduction

Ecologists use a large array of root functional traits to study plants (Freschet et al., 2021a,b). Root density (i.e., root biomass per volumetric unit of soil) is an important but often neglected plant trait that contains information about the resource investment of plants into foraging belowground (Cabal et al., 2021a). Understanding how plants allocate biomass belowground is important in the context of climate change for a precise assessment of carbon storage in plants (Xia et al., 2017; Qi et al., 2019) and for a more efficient food production (Anten and Vermeulen, 2016; Fréville et al., 2019). Root allocation strategies in plants are complex (McNickle et al., 2016) and may be based on a combination of various abiotic (resources in soil) and biotic (root detection, inter-plant communication, and soil microorganisms) information sources (Novoplansky, 2019; Chen et al., 2020). As a result of such complexity, different plant species may invest more or less into their roots as a response to the presence of non-self roots in the soil (Belter and Cahill, 2015; Postma et al., 2021).

Over the last two decades, the Root Tragedy of the Commons (RToC)—initially a concept with potential to explain fine root density in competitive contexts—has become a controversial idea. Inspired by Donald (1968)’s plant ideotypes, Zhang et al. (1999) published the first game theory model of plant root proliferation as a response to competition resulting in root growth redundancy: plants in their model could increase their yield by reducing root growth. They named this phenomenon an RToC based on Hardin (1968)’s theory. Shortly after, Gersani et al. (2001) observed higher root density at the expense of yield in plants sharing rooting volume with conspecific neighbors as compared with plants growing solo, seemingly validating the RToC experimentally. Root ecologists first replicated their owned/shared experimental design finding similar results (Maina et al., 2002; Falik et al., 2003; O’Brien et al., 2005). Yet, later publications questioned the empirical evidence of an RToC alleging problems with Gersani’s owned/shared design (Laird and Aarssen, 2005; Schenk, 2006; Hess and De Kroon, 2007; Semchenko et al., 2007; Chen et al., 2012, 2015, 2020; McNickle, 2020) (see Box 1).

**Box 1. Gersani’s classic-owned/shared experimental design: A defense**

Owned/shared systems were used by Gersani et al. (2001) to produce control and interaction setups with constant total soil volume and nutrients available at the community level. The first limitation of this experimental design was identified by Laird and Aarssen (2005), who noticed that, because the intermingled roots in the shared container are weighted in bulk, owned/shared experimental designs can identify a spurious relationship between the shoot-to-root biomass ratio and the RToC because of the size inequalities leading to an aggregation bias. The confounding effects of changing volume and nutrients available to the plant with neighbor presence were highlighted shortly after (Hess and De Kroon, 2007; Semchenko et al., 2007) triggering most subsequent controversy (see McNickle, 2020 for a comprehensive review). According to these critiques, nutrients can be a confounding factor because, in the interaction treatment, the neighbors will deplete resources leading to lower resource concentration than in the control treatment. To control for this, they suggested keeping the amount of resource available per capita constant across treatments. Soil volume is a confounding factor because increasing rooting volume may promote an increase in root proliferation. Unlike the case of nutrients, rooting volume is not a fungible resource, i.e., all the soil volume is available to all the plants; hence, in the interaction treatment each plant has access to twice the soil volume than in the control. The combination of both factors is found particularly difficult to deal with, and in order to get around these problems researchers have suggested complex designs changing the pot volume and nutrient concentration (Chen et al., 2015), and have developed complex methods to analyze the resulting data (McNickle and Brown, 2014; Chen et al., 2020; McNickle, 2020). In this box, I will support the original approach of Gersani et al. (2001). I defend that the handling of nutrient availability and soil volume in classic owned/shared systems does not represent a problem to experimentally identify an RToC, because the control is not meant to represent the behavior of a plant alone but rather a way to estimate optimal root density.

**A. Nutrient availability:**

Game-theory RToC models are based on basic exploitative plants (Zhang et al., 1999; Gersani et al., 2001; Zea-Cabrera et al., 2006). Take the seminal model by Gersani et al. (2001)’s resource net gain (G) for a focal plant (*i*):

(1) $$G_{i}\left(u_{i},x\right)=\frac{u_{i}}{x}H\left(x\right)-C\left(u_{i}\right)$$

where *u*_*i*_ is the root density of each individual plant, *x* is the total root density so that $x=\sum_{i=1}^{n}u_{i}$, *H* is a saturating function that yields the total amount of resources taken up by all *n* plants in soil (hence, the first term in the equation yields the resources uptaken by the focal plant), and *C* is a cost function. Because each plant optimizes its own net gain selfishly (∂*G*/∂*u*_*i*_ = 0), plants in the model adjust their root proliferation to the resource net gain exclusively. Hence, plants engage in an RToC based on the exploitative information only. Indeed, a similar game theory model shows that plants not only increase their root density when a neighbor is present locally (Cabal et al., 2020), but also solo plants will increase their root density identically if the rate of the physical loss of resource rises (Cabal et al., 2021b). The RToC is an exploitative response, which means that it is based in the quasi-equilibrium conditions of the resources entering and exiting the soil, including the resource depletion caused by non-self roots. Adding supplementary resources in the interaction treatment of the owned/shared experiments to compensate for the neighbor-induced resource depletion overrides any possible evidence for an RToC, because it would cancel the mechanisms that trigger the RToC.

**B. Soil volume:**

The concerns regarding rooting volume based on the idea that each plant has access to twice the rooting volume in the interacting treatment are not justified, because in the owned/shared experiments testing for an RToC, the rooting volume is kept constant across treatments at the community level. Gersani et al. (2001) analyzed their root tragedy of the commons (RToC) model in a particular scenario in which both the total number of plants (*n* = 10) and the total rooting volume available to these plants were fixed. They considered three particular cases: (*N* = 1) the soil is partitioned in 10 equal compartments, with each plant having access to one of them (equivalent to their control treatment); (*N* = 2) the soil is partitioned in five equal compartments shared by pairs of plants (equivalent to their interaction treatment); and (*N* = 10) the soil is not partitioned, with all plants having access to all the rooting volume. The value of *N* indicates the number of plants sharing each soil partition. A formal definition to the *H*(*x*) and *C*(*u*) functions from Eq. 1 should first be established to be consistent with the equations that Gersani et al. (2001) may have used, with their graphical results as a reference. Let us assume that the nutrient uptake function *H*(*x*) is a saturating function of the following form:

(2) $$H\,\left(x\right)=\frac{\varphi -\varphi \,e^{-\theta \,x}}{\theta }\,$$

and the cost function is a quadratic equation of the following form:

(3) $$C\,\left(u\right)=\,\alpha \,u^{2}+\beta \,u\,$$

As the ESS is the solution that, if adopted by all coexisting plants—u* = *u*_*i*_ for any *i*—maximizes the resource net gain with respect to *u*_*i*_, and must satisfy ∂*G*/∂*u*_*i*_ = 0, we can write

(4) $$\frac{N-1}{N}\frac{\left(\text{φ}-\text{φ}e^{-\text{θ}nu^{*}}\right)}{\text{θ}nu^{*}}+\frac{1}{N}\text{φ}e^{-\text{θ}nu^{*}}=2\text{α}u^{*}+\text{β}$$

Using these equations, we can accurately reproduce all the results from Gersani et al. (2001). Unfortunately, there is no analytical solution that can be derived for u* in Eq. 4. But, we can approach the solutions numerically for the parameter values of φ = *0.3*, θ = *0.25*, α = *0.025*, and β = *0.025*, reproducing the authors’ original results, obtaining for the control treatment$\,u_{N=1}^{*}\approx 2.6181\,\,U\,n\,i\,t\,s\,o\,f\,R\,o\,o\,t\,\left(R\right)$, the interaction treatment$u_{N=2}^{*}\approx 3.0174\,\,R$, and the ten plants sharing a soil volume$u_{N=10}^{*}\approx 3.3959\,\,R$. These values represent the total amount of roots one plant produces in the total soil volume.

Model results do not control for rooting volume in the manner assumed by researchers (i.e., accounting for the rooting volume actually available for each plant in each “pot”). However, this can be calculated by defining a “pot” or unit of rooting volume (*v*) as a tenth of the total soil volume in the model. Thereafter, *m* can be defined as the number of rooting volume units per compartment, while the plant root density *d* can be defined as the units of root for each plant in each soil compartment (*R*/*v*). We can calculate each plant’s root density in equilibrium using:

(5) $$d^{*}=\frac{u^{*}}{m}$$

Although root production per plant in the total soil volume increases with competition intensity (*N*), the root density per plant actually decreases; for the control treatment, root density per plant is $\,d_{N=1}^{*}\approx 2.618\,\,R/v$, and for the interaction treatment, this value is$d_{N=2}^{*}\approx 1.509\,\,R/v$. Nonetheless, such result does not mean that plants are not engaging in an RToC. The optimal collective rooting strategy x* satisfying*dG*_*T*_/*dx* = 0 (the optimal root proliferation for any amount of plants sharing a unit of soil volume), where:

(6) $$G_{T}\left(x\right)=H\left(x\right)-C\left(x\right)$$

is equivalent to the optimal root production of a plant owning a unit of soil volume when plants follow the strategy u* satisfying *dG*_*i*_/*du*_*i*(*N* = 1)_ = 0. This equality indicates that the maximum collective gain is reached when the root production per plant across all soil space is equivalent to the root production of plants in individual soil compartments. In general, this equality indicates that, given the choice of parameters, a total root density of 2.6181 *R*/*v* is optimal, regardless of the number of plants growing roots. Collective gain is optimized at this density, as confirmed by plugging the values in the resource net gain equation: the collective net gains are G_*N=1*_ = 0.3396, G_*N=2*_ = 0.2333, and G_*N=10*_ = 0.2032 resources per plant. In the *N* = 2 and *N* = 10 scenarios, plants are engaging in an RToC by overproliferating their roots with respect to the collective optimal, and are thus inefficiently overexploiting the common resource.

Gersani et al. (2001)’s experiment, and similar owned/shared designs, have been criticized because researchers have interpreted that the root allocation of a plant growing alone in one pot was compared to the root allocation of a plant sharing two pots with a neighbor. The key to understand this type of experimental design relies on correctly interpreting how their model was constructed, and realizing that their control treatment is a proxy for optimal collective root densities. The fundamental assumption of this design is that non-competing plants forage optimally (i.e., they do not have a root overproliferation fixed by an evolutionary arms race), hence, one can calculate the optimal root density (root biomass per unit volume or pot) that maximizes net gain, whether one or several plants share the pot. This root density serves as a base to estimate collective optimal in the two-pot system: If the root density in shared pots is higher, researchers can deduce that competing plants engage in an RToC (Figure Box 1).

**Figure Box 1:**
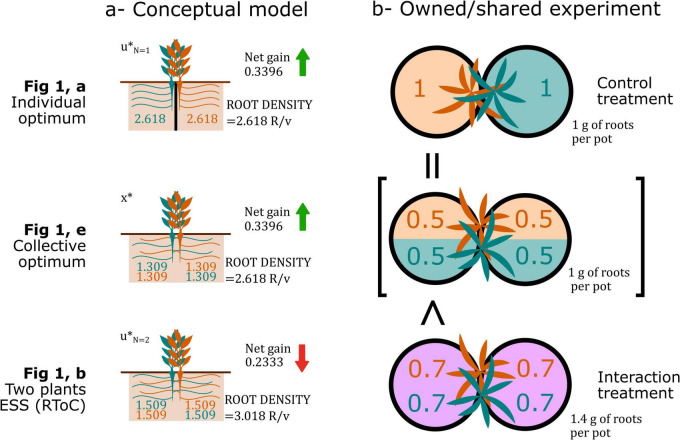
Results from Gersani et al. (2001), left, conceptual model (values based on the numerical results shown in Box) and, right, experimental results (approximated values from the original paper’s results) depicting how the individual optimal is analogous to the collective optimal of the two plants sharing two soil volumes in their approach.

The ecology of plant interactions is currently moving toward an approach centered on individual plants and their phenotypical plasticity (Bakker et al., 2021; Escudero et al., 2021) but there is very little we know about how plant interactions affect the plastic response of plants to abiotic conditions (Wang and Callaway, 2021). Accounting for the RToC represents a mechanistic approach to the study of phenotypically plastic responses of plants to neighbors, because game theory produces mechanistically informed dynamic predictions. To promote this approach, I briefly revisit the literature from a critical perspective and sticking to the original definition of a “tragedy of the commons” (*sensu* Hardin, 1968). I argue that, while researchers who question the RToC have focused on experimental flaws, there is an underlying dissent between their use of the RToC and Hardin’s definition. They treat the RToC as a proactive competitive strategy, while it is an inevitable consequence of the simplest optimization of resource foraging. This confusion has led to the disparagement of a theory that can be crucial to interpret experimental results correctly and to better understand root-density phenotypic plasticity in the plants.

## Discussion

### Defining the root tragedy of the common

Hardin (1968) defined the tragedy of the commons using the famous example of the shared pasture, the herdsmen, and the cattle. In this example, each herdsman finds a net reward in adding one more head to his herd above the optimal cattle density, because the benefits from the animal are for the herdsman alone but the community shares the costs of the decreased pasture quality. The herdsman does not need to do this calculation; he will just realize that adding one more animal is profitable. Since all the herdsmen act the same way, they will overexploit the common resource unwittingly, to the detriment of all of them. In a plant root analogy, each individual of plant represents a herdsman, each root unit is like an animal that forages at a cost, and the soil resource plays the role of the pasture.

**FIGURE 1:**
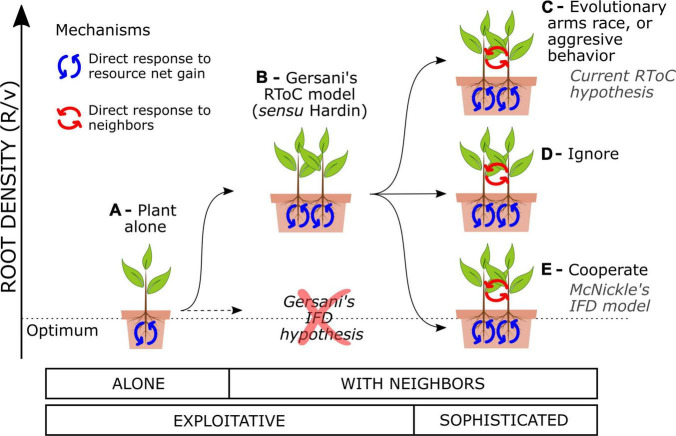
Collective root density in soil as a function of varying the interaction setup and the type of plants. **(A)** An exploitative plant alone may tend to an optimal root density in equilibrium with the resource dynamics. **(B)** Interacting exploitative plants engage in an RToC (*sensu* Hardin). Sophisticated plants can directly detect their neighbors and tune their exploitative response in any direction: **(C)** aggressively, overproliferating roots above the RToC (called also an RToC in modern studies), **(D)** ignoring their neighbors in terms of root density, or **(E)** cooperating and approaching and optimal collective root density (sometimes called an IFD in modern studies).

Plants that are programmed to forage optimally for soil resources disregarding the presence of the neighbors will engage in an RToC. Plants foraging strategies consist in adjusting their root density to the environment through evolutionary fixed traits or phenotypic plasticity. Plants display a large phenotypic plasticity in fine root proliferation (Callaway et al., 2003; Kembel and Cahill, 2005) and will adjust their root density over their lifespan based on both the abiotic information (Hodge, 2004) and the presence of competing neighbors (Craine, 2006). I will herein assume that the most basic plant response is the exploitative response to the abiotic environment, i.e., plants that adjust their root growth to the net resource gain that such roots return (Figure 1A). For instance, an imaginary plant species that had evolved growing with no neighbors for millions of years in heterogeneous soils should become exploitative. Exploitative plants still respond to the presence of neighbors, because the neighbors modify the resource dynamics in soil and the plant must adjust its root density accordingly (Craine, 2005; O’Brien and Brown, 2008; Pierik et al., 2013). Game theory models that assess the response of the purely exploitative plants to the presence of neighbors predict that such plants must engage in an RToC (Zhang et al., 1999; Gersani et al., 2001; Cabal et al., 2020). Gersani et al. (2001) hypothesized that competing exploitative plants could either downregulate root growth to keep collective root density constant as plant population density increases (an “ideal free distribution,” IFD) or follow game theory model predictions and overproliferate roots with respect to the collective optimum (RToC)—not necessarily to the plant alone (Kim et al., 2021). They used the IFD as a null hypothesis to test against the RToC in their experiments with soybeans, finding empirical support for the RToC (Figure 1B).

Plants that have developed a direct response to the presence of neighbors, i.e., that are competing proactively, may use adventitious decision-making algorithms to tune their exploitative response to the present neighbors (hereafter “sophisticated plants”). Evolutionary ecology gives examples of plants evolving fixed suboptimal strategies as a direct response to competitors (Rankin et al., 2007), such as the case of the evolutionary arms race in competition for light which leads to trees investing in trunk wood (Falster and Westoby, 2003; Dybzinski et al., 2011). Also, many publications explicitly state or implicitly assume that plants engage in an RToC when they overproliferate roots as a response to direct self/non-self root discrimination (see for instance, Hess and De Kroon, 2007; Padilla et al., 2013; Chen et al., 2015, 2020; McNickle, 2020), a strategy that could be called a root aggressive behavior. Both hypotheses, the root arms race and the root aggressive behavior, are reasonable, but neither of them represents an RToC (Figure 1C). Sophisticated plants could also reduce their root density with respect to the exploitative RToC as a response to neighbor presence, either behaviorally or through evolutionary fixed strategies. McNickle and Brown (2014) call such strategy an IFD in their model of plants engaging in collective optimal root foraging strategies (Figure 1E). While understanding both the exploitative and the sophisticated responses of plants has an intrinsic value, the use of the same terminology (RToC and IFD) to name different mechanisms driving plant responses has led to confusion in the field.

According to game theory, only sophisticated plants—not purely exploitative ones—can pursue a collective optimal strategy. Game theory models suggest that the active recognition of other stakeholders and the implementation of complex mechanisms is actually necessary to avoid a tragedy of the commons (He et al., 2015; Murase and Baek, 2018). Likewise, the sophisticated plants need to gather information (other than detecting the mere presence) about each other in order to avoid the RToC and engage in an IFD *sensu* Gersani et al. (2001). This is conspicuous when assessing the cooperative solution of game theory root competition models: the optimization conditions for every individual depend on the net-gain-generating equations of all the coexisting individuals (Pulliam et al., 1982; McNickle and Brown, 2014; Cabal et al., 2020). Accordingly, plants showing an optimal root density when interacting with each other must be able to measure the total resource net gain of non-self roots in the shared soil. Plants must be unable to avoid engaging in an RToC if they lack the physiological capacity to gather complex information about non-self roots, such as how efficient they are foraging resources. This represents a challenge for maximizing yield in crops by means of controlling root growth (Schneider and Lynch, 2020).

### Identifying the root tragedy of the common

Experimental designs, and in particular, Gersani et al. (2001)’s owned/shared setup, have had a central role in the RToC controversy. The owned/shared experimental design consists in a control treatment in which a plant owns a unit of soil volume (a pot or compartment), and an interaction treatment in which two plants share two units of soil volume. It represents a very convenient experimental design due to its simplicity. Criticisms to this experimental setup are the basis of most studies questioning the classic RToC, but there are good reasons to believe the design is actually correct (see Box 1).

Alternatively, the mesh divider experimental design has become popular (McNickle, 2020) and is often used today to test for the RToC (Zhu et al., 2019, 2020; Chen et al., 2021). In a mesh divider setup, each plant owns a partition of a container. Partitions are separated by a permeable mesh in the interaction treatment, whereas in the control treatment, the separation is not permeable. Because resources would only flow across the mesh if diffusion is driven by a nutrient concentration or a water potential gradient (Kirkham, 2014) and plants in each compartment are typically identical, both the compartments must be symmetrical in resource concentration distribution and no force will trigger resource mixing despite the permeability of the mesh. Therefore, the interaction treatment does not differ from the control treatment in terms of soil resource. Contrastingly, other chemical substances will diffuse freely from one plant’s to another’s partition (Kong et al., 2018). Hence, mesh divider systems test the effect of non-resource mechanisms controlling for the exploitative response.

When owned/shared experiments detect a root overproliferation, it may be a purely exploitative response, or it could be the combined result of the RToC, an aggressive strategy, and/or a root arms race. Isolating each phenomenon is crucial to understand mechanistically the plant-foraging strategy.

Isolating the aggressive behavior from the RToC consists in measuring to what extent the root overproliferation detected in an owned/shared experiment is triggered by direct non-self roots detection. It is possible to measure that by complementing owned/shared with mesh divider experiments. For instance, Gersani et al. (2001) found root overproliferation in their owned/shared experiment with soybean plants (*Glycine max*) while Chen et al. (2021) found no response in a mesh-divider experiment. The former study demonstrates that soybean plants overproliferated roots with respect to the optimal root density when they interact, yet the latter indicates that this species did not respond directly to the neighbor detection. In conclusion, soybean plants seem to engage in an RToC as classic papers pointed out. Because the fundamental effect of non-self roots on resource dynamics is to increase resource depletion (Schenk, 2006), I suggest growing plants alone and vary the resource decay rate as an alternative experimental design to investigate the purely exploitative response of plants. In this setup, resource decay would emulate non-self root resource depletion without the non-self roots being actually present. While researchers have experimentally assessed the response of plants to soil patches with different resource availability, this is typically done by controlling for resource inputs only (Hodge, 2004). However, the response to resource availability may be quite different when the changes in availability are driven by the inputs or decay rates, only the latter being analogous to depletion by the neighbors (Cabal et al., 2021b).

Isolating the evolutionary root arms race from the RToC consists in determining to what extent any plant, even when growing alone, is overproliferating roots as a fixed trait. On the contrary, this may not be considered a confounding factor in owned/shared experiments because the difference between solo and interacting plants may remain unchanged (the fixed root proliferation happens in both the cases). However, determining whether plants engage in a root arms race seems more challenging than identifying behavioral responses, because we lack manageable control treatments. A possible experiment would consist in performing owned/shared experiments on both the wild plants and their respective cultivars and compare their responses, because we could expect domestic varieties to be bred to avoid an arms race, and maybe also to attenuate the RToC.

## Conclusion

Modern studies consider the RToC as the case in which plants, actively detecting their neighbors, overallocate resources into their roots compared with the plants growing alone. Nevertheless, the classic RToC *sensu* Hardin happens when plants invest more into their roots than the community-level foraging optimal based on the information about soil resource dynamics. While sophisticated strategies based on neighbor detection mechanisms may override and mask the RToC in some species, we must see the RToC as a baseline exploitative response of all the plants to the interaction with neighbors. Classic owned/shared experiments are a convenient design to identify the trace of an RToC in exploitative plants, but other complementary experiments such as mesh divider systems, varying abiotic resource decay rates, or comparing wild species and their relative cultivars, can provide valuable information to isolate the effects of other mechanisms and phenomena at play.

## Author contributions

The author confirms being the sole contributor of this work and has approved it for publication.

## Glossary

*Exploitative plant*: A plant or plant species which belowground foraging strategy is based exclusively in maximizing resource net gain, i.e., maximizing the difference between the resource uptake by roots and the cost of building and maintaining such roots, and adjust its root production accordingly.

*Sophisticated plant*: A plant or plant species that can collect more environmental information than an exploitative plant, e.g., self/non-self root discrimination, and use that information to tune its exploitative foraging strategy.

*Self/non-self root discrimination*: The ability of a root to determine whether other surrounding roots are connected to self by active vessel transportation (i.e., belong to the same individual plant) or not.

*Tragedy of the commons sensu Hardin (1968)*: A strategy predicted by game theory models in which several stakeholders sharing a common resource, each adjusting their resource-use strategy only aiming to maximize its net reward (=benefit/uptake - cost/investment), unintentionally and inevitably overexploit the resource to the detriment of all.

*Evolutionary arms race sensu* Dawkins and Krebs (1979): A strategy predicted by game theory models in the context of evolutionary ecology in which individuals evolve “weapons” (i.e., adaptations designed to harm or outcompete neighbors), engaging in an apparently unlimited escalation of the allocation of resources to such weapons in the course of evolution.

*Root aggressive behavior*: A strategy in which individuals proliferate more roots than exploitative plants when they encounter non-self roots in the soil, as a pre-emptive strategy to deplete soil resources and outcompete the neighbors.

Ideal free distribution (IFD) *sensu* Fretwell and Lucas (1969): A strategy predicted by bird territoriality models in which ideal free individuals adjust through migration the population density within each habitat so that the suitability in all habitats, which decreases as occupancy increases, remains equal.

*—sensu* Gersani et al. (2001): They extrapolated this concept to root competition, defining it as a passive habitat selection that leads to no difference in root proliferation or reproductive yield between owners and sharing individuals. This is to be interpreted in the context of their paper, therefore, plants producing as many roots in one soil compartment when owning it as in N soil compartments when sharing them with N plants (hence 1/N times roots per compartment).

—*sensu* McNickle and Brown (2014): They revisited the concept, but described it as a case in which plants grow roots in proportion to nutrient’s availability at each soil location but do not have any direct response to neighbors that is independent of their response to nutrients (the features actually leading to an RToC!). Nevertheless, their IFD model incorporates cooperative behaviors based on the complex neighbor information (the optimization conditions for every individual depend on the net-gain-generating equations of all the coexisting individuals).
